# Distribution areas and monthly dynamic distribution changes of three *Aedes* species in China:* Aedes aegypti*, *Aedes albopictus* and* Aedes vexans*

**DOI:** 10.1186/s13071-023-05924-9

**Published:** 2023-08-26

**Authors:** Yuepeng Li, Qi An, Zhuo Sun, Xiang Gao, Hongbin Wang

**Affiliations:** 1 College of Veterinary Medicine, Northeast Agricultural University, Harbin, People’s Republic of China; 2 Key Laboratory of the Provincial Education, Department of Heilongjiang for Common Animal Disease Prevention and Treatment, College of Veterinary Medicine, Northeast Agricultural University, Harbin, People’s Republic of China

**Keywords:** *Aedes aegypti*, *Aedes albopictus*, *Aedes vexans*, Monthly distribution variation, Ecological niche model

## Abstract

**Background:**

Mosquitoes play an absolute role in the spread of epidemic arbovirus diseases. Worldwide, *Aedes aegypti* and *Aedes albopictus* are the main vectors responsible for the spread of these mosquito-borne diseases. *Aedes vexans*, a mosquito species native to China, also carries mosquito-borne viruses, such as dengue fever virus and Japanese encephalitis virus, but research on this mosquito has been inadequate. Mapping the potential distribution range of and monthly change in the distribution of these three *Aedes* species is of particular importance for mosquito surveillance, eradication and disease control.

**Methods:**

Monitoring data were collected for the three *Aedes* species in China. Long-term temperature and precipitation data (2001–2021) and land cover data were used to represent various climate and environmental conditions. An ecological niche model was developed using a maximum entropy modeling method to predict the current optimum habitat areas for the three *Aedes* species and to obtain important variables influencing their monthly distribution.

**Results:**

The distribution model for the three *Aedes* species performed well, with an area under the receiver operating characteristic curve value of 0.991 for *Ae. aegypti*, 0.928 for *Ae. albopictus* and 0.940 for *Ae. vexans*. Analysis of the distribution change and mapping of the optimum habitat range for each *Aedes* species for each month demonstrated that temperature, precipitation and construction land were important factors influencing the distribution of these three *Aedes* species.

**Conclusions:**

In China, *Aedes aegypti* is mainly concentrated in a few tropical regions and along the Yunnan border; *Aedes albopictus* is widely distributed throughout most of the country, except for the arid and semi-arid regions of northwest China; and *Aedes vexans* is mainly found in the northern regions. Our results provide a basis for the timing and location of surveillance efforts for high-priority mosquitoes.

**Graphical abstract:**

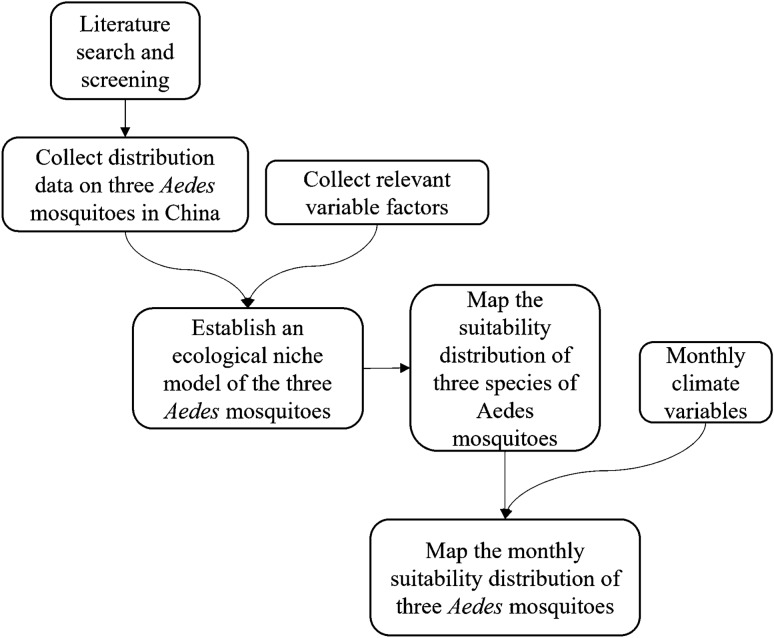

## Background

Arbovirus disease is a generic term for a group of diseases caused by specific viruses. These viruses are transmitted to humans or animals through the bite of an infected arthropod. Mosquitoes are one of the most important species that spread these viruses. As an arthropod, mosquitoes are taxonomically divided into 41 genera and about 3500 species in the family *Culicidae* [1]. The most notable mosquito-vector diseases are Zika virus disease [2], dengue [3], yellow fever disease [4], chikungunya virus disease and Japanese encephalitis, all of which are transmitted by mosquitoes of the genus *Aedes* [5]. *Aedes albopictus* and *Aedes aegypti* are the main vectors for the transmission of these viruses [6, 7]. While members of the mosquito genus *Culex* are considered to be the primary vector of Japanese encephalitis, there is evidence that *Ae. vexans* is responsible for a significant amount of the transmission of this disease, supported by the isolation of Japanese encephalitis virus (JEV) from *Ae. vexans* [8–10].

Between 1978 and 1991, dengue fever was mainly reported in the provinces of Guangdong and Hainan; after 1990, the dengue fever epidemic in China expanded to the provinces of Guangxi, Fujian, Zhejiang, Jiangsu, Yunnan, Henan and Beijing [1]. *Aedes aegypti* and *Ae. albopictus* are the main vectors of dengue fever transmission [11]. *Aedes aegypti* can be more effective in transmitting dengue fever, and historically most dengue outbreaks in China were caused by *Ae. aegypti* [12, 13]. *Aedes aegypti* was originally found only in tropical cities below 22°N latitude in Hainan, Guangxi and Guangdong [14], but its range has expanded with the changes in the climate [15], and since 2002, *Ae. aegypti* has been found along the border areas of Yunnan province [16]. *Aedes albopictus* is distributed in temperate, subtropical and tropical regions of China south of 41°N latitude, mainly in peripheral urban and rural areas [17].

While *Ae. aegypti* and *Ae. albopictus* have been repeatedly identified as transmitting many of the prevalent mosquito-borne diseases, the presence of *Ae. vexans* has been overlooked. While *Ae. vexans* is not considered to be a vector of Japanese encephalitis, JEV has been detected in *Ae. vexans* [18], suggesting that it plays an important role in the transmission of the disease. In addition, Tahyna virus [19], Banna virus [1], Getah virus [20] and Chaoyang virus [21], all of which occur in China, can be transmitted by *Ae. vexans*. *Aedes vexans* is a mosquito native to China where it is widely distributed. It is found as far north as Heilongjiang province, as far east as the Shandong Peninsula, as far south as Yunnan province and as far west as Xinjiang province, occupying most of the country, but primarily distributed in the northern regions of China [22]. The West Nile virus can be transmitted to mice by *Ae. vexans* under laboratory conditions, as demonstrated by Anderson et al. [23]. *Aedes vexans* is the dominant mosquito species in the three northeast provinces of China and northwest China, and it has a high density in the border areas of Xinjiang [24].

Mosquito monitoring mechanisms and data compilation on mosquito vectors in China are not perfect at the present time. However, for mosquito surveillance, eradication and disease control, it is necessary to be able to provide complete reports on mosquito suitability distribution and a monthly dynamic distribution map. In this study, three important *Aedes* species, each with its own characteristic geographical distribution, were selected as research objects. In China, *Aedes aegypti* is only distributed in the tropical regions and along the border in Yunnan, whereas *Ae. albopictus* is spread over most of southern China, across the three climate zones of the country. *Aedes vexans* is spread throughout China and is the dominant mosquito species in northern China, carrying various mosquito-borne viruses [1], transmitting many mosquito-borne diseases and facilitating the geographical transmission of potentially dangerous viruses [22].

This study reported here applies ecological niche modeling to investigate the effects of climate and environmental changes on the distribution of these three *Aedes* species, analyze their optimum temperature and precipitation ranges and map their current suitability distribution and monthly dynamic distribution. The results will provide a basis for their management and for the prevention and control of mosquito-borne diseases, as well as basic information for ecological studies of the genus *Aedes*.

## Methods

### Species data collection

The three *Aedes* species collected in this study were *Ae. aegypti*, *Ae. albopictus* and *Ae. vexans*. The data points of these *Aedes* species were obtained from three sources. First, a comprehensive search of the literature was performed using databases, including the China Knowledge Network, Google Scholar, Web of Science and PubMed. Articles that provided detailed information on sampling locations or latitude and longitude coordinates were used, and only records from 1990 up to the present were included. Second, data were obtained for China from the global database of *Ae. aegypti* and *Ae. albopictus* compiled by Kraemer et al. [25] (http://dx.doi.org/10.5061/dryad.47v3c). Third, records of the three *Aedes* species were obtained for mainland China from the Global Biodiversity Information Facility (GBIF) database (https://www.gbif.org/), which contains global occurrence records for many species. The ENMTool software was used to filter duplicates of collected *Aedes* species data and reduce spatial autocorrelation, ensuring that only one data point was included in each raster cell. Finally, 97 *Ae. aegypti* data points, 444 *Ae. albopictus* data points and 133 *Ae. vexans* data points were used for subsequent model construction.

### Climate and land cover data

Climate and land cover variables included temperature, precipitation, land cover fraction and elevation. Temperature, precipitation and land cover fraction datasets were downloaded from the National Tibetan Plateau Data Center (https://data.tpdc.ac.cn/zh-hans/data/ee4de140-1c2e-4e30-866b-5a6f5c57e3bb). We obtained mean monthly temperature and precipitation data from January 2001 to December 2021, and calculated the mean annual temperature and precipitation for each of the 21 years to represent current temperature and precipitation. The land cover fraction dataset provides eight land cover types in China, including forest, grassland and shrub (GS), cropland, wetland, water body, construction land, bare land and permanent snow and ice (PSI), with a time series from 2001 to 2018. The annual average for each land cover type was calculated for subsequent modeling. Lastly, Shuttle Radar Topography Mission (SRTM) elevation data were downloaded from the World Climate website (https://worldclim.org/), and elevation data for China were obtained using the mask extraction tool of ArcGIS (v10.2; ESRI, Redlands, CA, USA). The resolution of all variables used for modeling was set to 0.0083333° (i.e. approx. 1 × 1 km) (Table 1).

**Table 1:** Variables used in the model

Variable cord	Variable name	Source
bare_ land	Bare land	National Tibetan Plateau Data Center
construction	Land for Construction	National Tibetan Plateau Data Center
cropland	Cropland	National Tibetan Plateau Data Center
forest	Forest	National Tibetan Plateau Data Center
gs	Grassland and Shrub	National Tibetan Plateau Data Center
pre	Average annual precipitation/12	National Tibetan Plateau Data Center
psi	Permanent Snow and Ice	National Tibetan Plateau Data Center
tmp	Average monthly temperature	National Tibetan Plateau Data Center
water	Water	National Tibetan Plateau Data Center
wetland	Wetland	National Tibetan Plateau Data Center
elev	Elevation	WorldClim version 2.1

### Ecological niche modeling

Maxent v3.4.1 (http://biodiversityinformatics.amnh.org/open_source/maxent/) is widely used for ecological niche modeling. Thus, this maximum entropy modeling method was used to predict the suitability distribution of the three *Aedes* species in China. The model was established as follows. The data points of *Aedes* species were divided into two parts: 80% as the training set and 20% as the test set, using a background-presence modeling approach; the regularization constant was set to 1 and the model was run 100 times. The area under receiver operating characteristic curve (AUROC) value was used to assess the predictive power of the model, with larger AUROC values indicating better predictive performance of the model. Finally, a binary suitability map was generated, with values close to 0 indicating that the three *Aedes* species were unsuitable for the areas, and values close to 1 indicating that the three *Aedes* species were suitable for the areas. ArcGIS mapping software was used to visualize the results.

### Monthly suitability distribution map for *Aedes* species

Monthly variations in temperature and precipitation were used to predict the monthly suitability distribution areas for the three *Aedes* species. Based on the model results, we combined the equal sensitivity and specificity threshold with the optimum temperature and precipitation ranges of the three *Aedes* species. The monthly temperature and precipitation datasets from January 2001 to December 2021 were selected to obtain 252 months of data, and the mean monthly temperature and precipitation from January to December for 21 years were calculated. These data were converted to a raster map in ArcGIS.

The appropriate range of mean monthly temperature and precipitation for each *Aedes* species was determined based on the response curves and the equal sensitivity and specificity threshold calculated from model iterations. Next, a raster calculator was used to obtain raster layers of the suitable range of mean monthly temperature and precipitation for each *Aedes* species from January to December, and the two raster layers were overlaid with the habitat suitability map; the intersection was taken to produce a habitat suitability map for each *Aedes* species from January to December.

## Results

### Current distribution of the three *Aedes* species

**Fig. 1:**
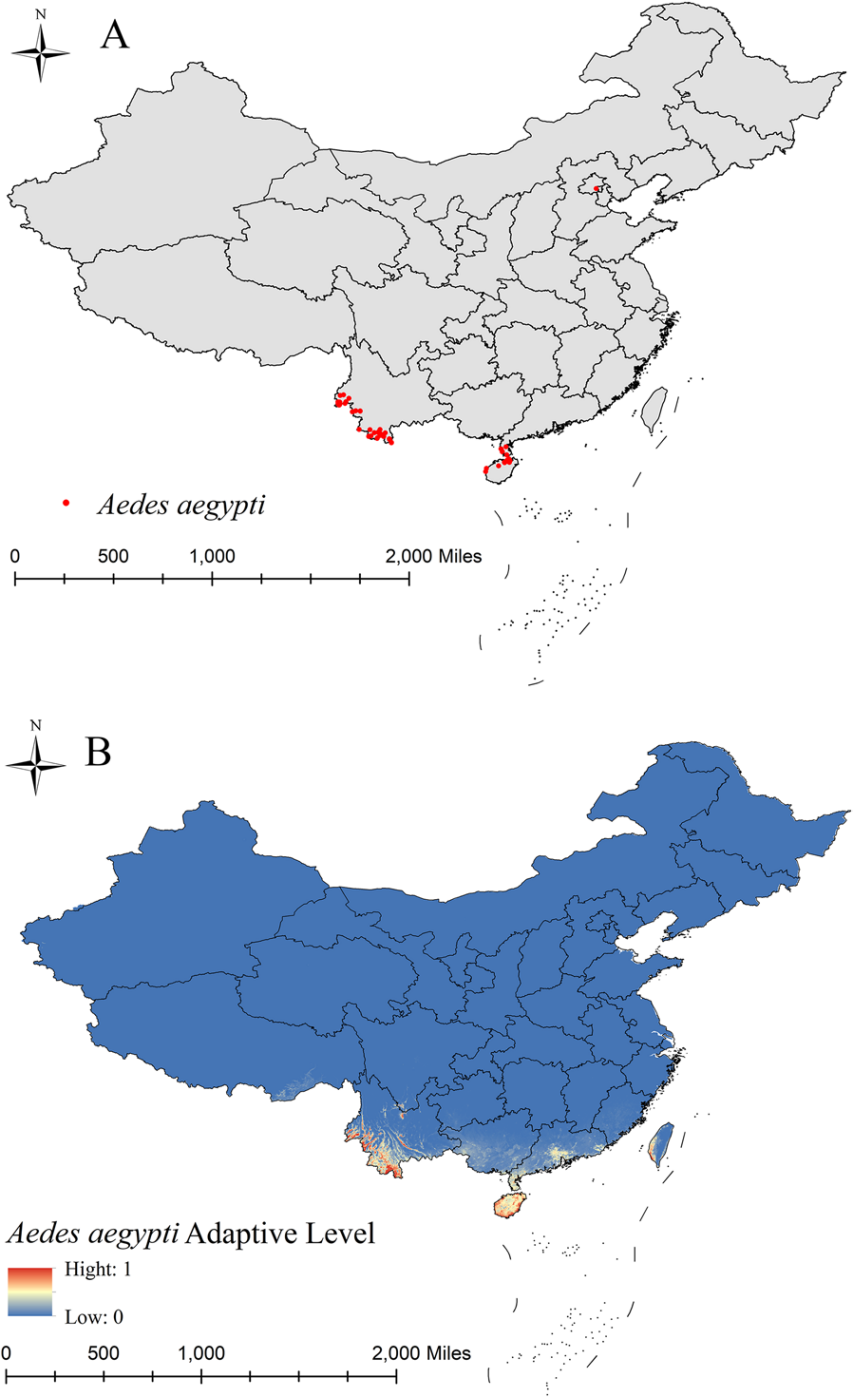
Surveillance records on *Aedes aegypti* distribution (**A**) and areas of suitability (**B**)

**Fig. 2:**
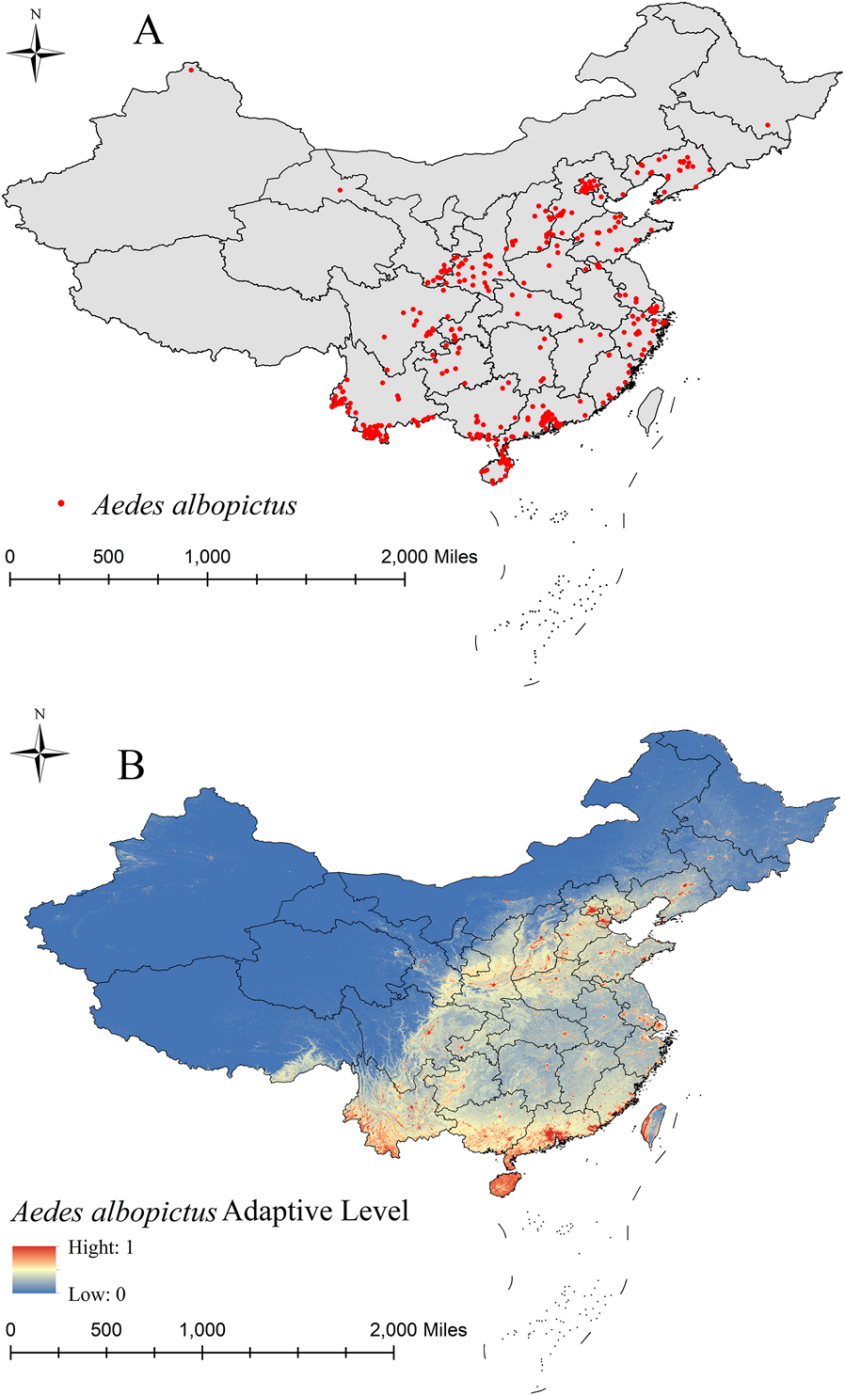
Surveillance records on *Aedes albopictus* distribution (**A**) and areas of suitability (**B**)

**Fig. 3:**
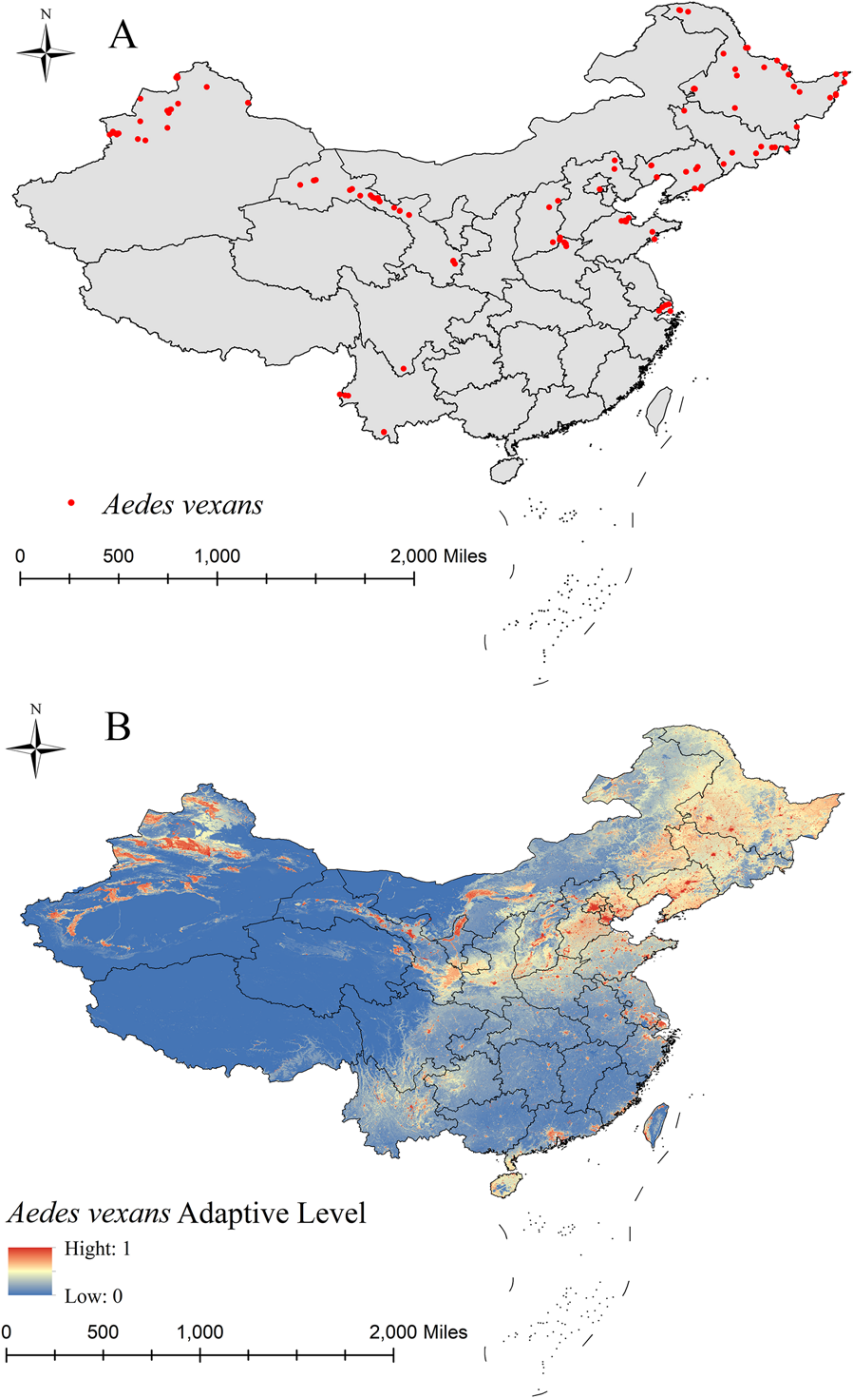
Surveillance records on *Aedes vexans* (**A**) and areas of suitability (**B**)

The data points of the three *Aedes* species were visualized and presented on a map of China using ArcGIS mapping software. The results indicated that the ecological niche model for the three *Aedes* species performed well, with an AUROC value of 0.991 for *Ae. aegypti*, 0.928 for *Ae. albopictus* and 0.940 for *Ae. vexans*; these values were good representations of the current areas of suitability for the three mosquito species. The suitability distribution range of *Ae. aegypti* was relatively limited, mainly to along the Yunnan border, Hainan, the Leizhou Peninsula in Guangdong and the southwest of Taiwan Province (Fig. 1). *Aedes albopictus* had a relatively widespread distribution, present over most of China south of 41°N latitude, with a distribution concentrated in southern, eastern and central China, and even parts of northeast China (e.g. Jilin) (Fig. 2). *Aedes vexans* was mainly found in northwest and northern China, Xinjiang and the three eastern provinces in China (Fig. 3).

### Variable importance analysis

A variable was considered to be important in influencing the distribution of a mosquito species if its variable contribution rate was > 10%. We found that precipitation, temperature and construction land were important factors influencing the distribution of these three *Aedes* species, but there were slight differences among the mosquito species. For *Ae. aegypti*, temperature and precipitation were important factors influencing its distribution, with variable contribution rates of 64.6% and 13.7%, respectively (Table 2). The distribution of *Ae. albopictus* was strongly influenced by construction land, precipitation and temperature, with variable contribution rates of 46.7%, 22.3% and 15.1%, respectively (Table 3). Construction land and precipitation had a greater influence on the distribution of *Ae. vexans*, with variable contribution rates of 45.8% and 12.2%, respectively (Table 4).

**Table 2:** Contribution and permutation importance of variable in the final models of *Aedes aegypti*

Variable	Percent contribution (100%)	Permutation importance (100%)
Tmp	64.6	91.7
pre	13.7	1.7
elev	9.9	5.2
construction	9.8	0.2
forest	0.7	0.1
bare_land	0.5	0.6
cropland	0.5	0
gs	0.3	0.5
water	0	0
wetland	0	0
psi	0	0

**Table 3:** Contribution and Permutation importance of variable in the final models of *Aedes albopictus*

Variable	Percent contribution (100%)	Permutation importance (100%)
construction	46.7	20
tmp	22.3	38.9
pre	15.1	22.2
bare_land	7	6
gs	3.7	3.3
elev	3.3	5.6
forest	0.8	1.7
cropland	0.7	1.6
water	0.2	0.6
wetland	0.1	0.1
psi	0	0

**Table 4:** Contribution and permutation importance of variable in final models of *Aedes vexans*

Variable	Percent contribution (100%)	Permutation importance (100%)
construction	45.8	13.7
tmp	12.2	12.9
elev	9.3	23.7
cropland	8.9	6.9
bare_land	8.5	17.5
gs	7.6	10.2
pre	4.4	9.5
forest	1.9	4.3
water	0.9	0.6
wetland	0.5	0.7
psi	0	0

### Monthly distribution changes in the three *Aedes* species

**Fig. 4:**
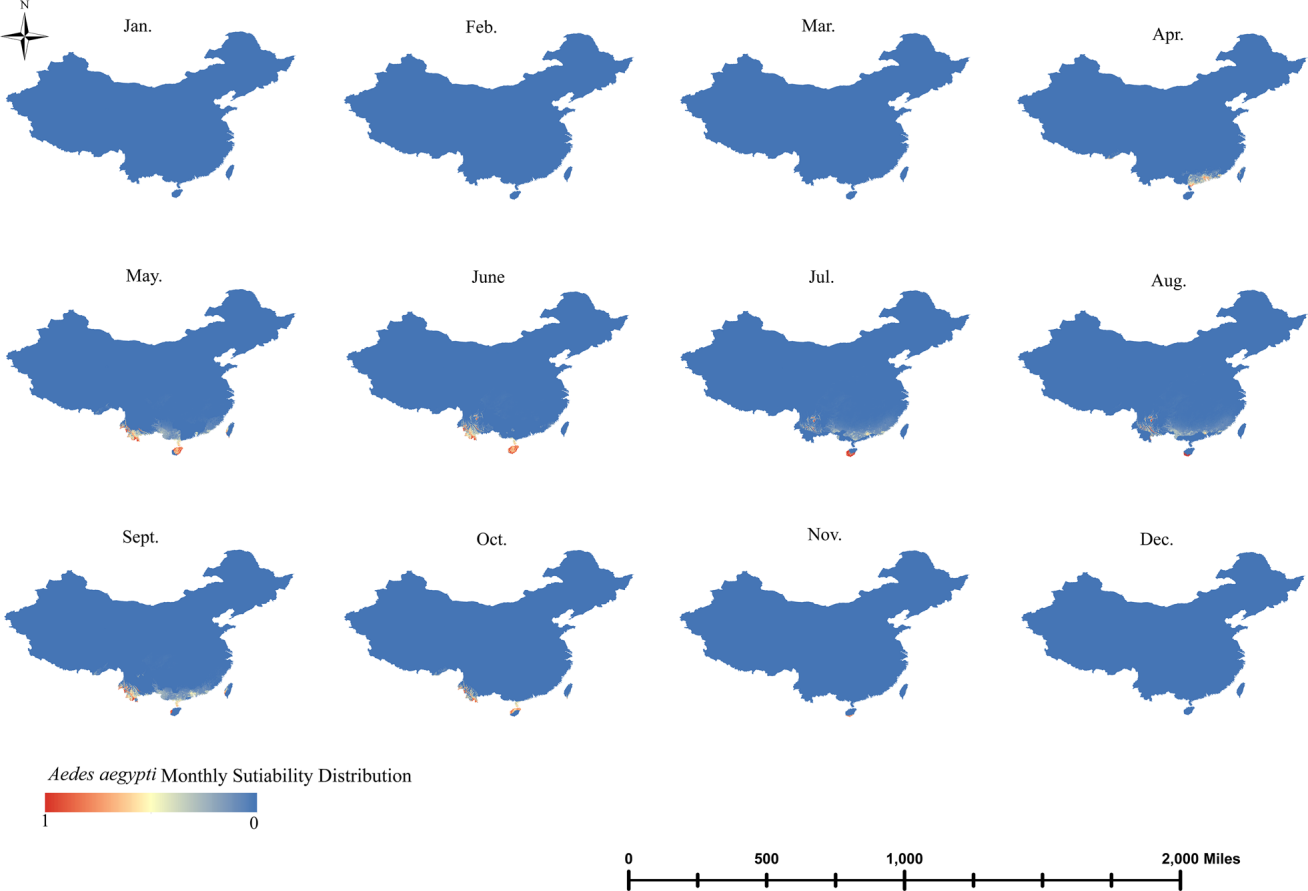
Monthly dynamic distribution range of *Aedes aegypti*

The monthly dynamic distribution map provides a good indication of variations in the monthly distribution of the three *Aedes* species from January to December. *Aedes aegypti* appeared from April until the end of October, with a more restricted distribution range in October. In April, *Ae. aegypti* was concentrated in the southern region of Guangdong, and in May and June, it was mainly concentrated in the border areas of Yunnan, the Leizhou Peninsula in Guangdong and Hainan, where the range of suitability distribution areas was the greatest. *Aedes aegypti* was mainly concentrated in Hainan in July and August, whereas the Yunnan border and southern Guangzhou areas were unsuitable for its distribution during these months. It reappeared at the Yunnan border in September and October, with the Leizhou Peninsula in Guangdong and parts of Hainan also showing high suitability at this time (Fig. 4).

**Fig. 5:**
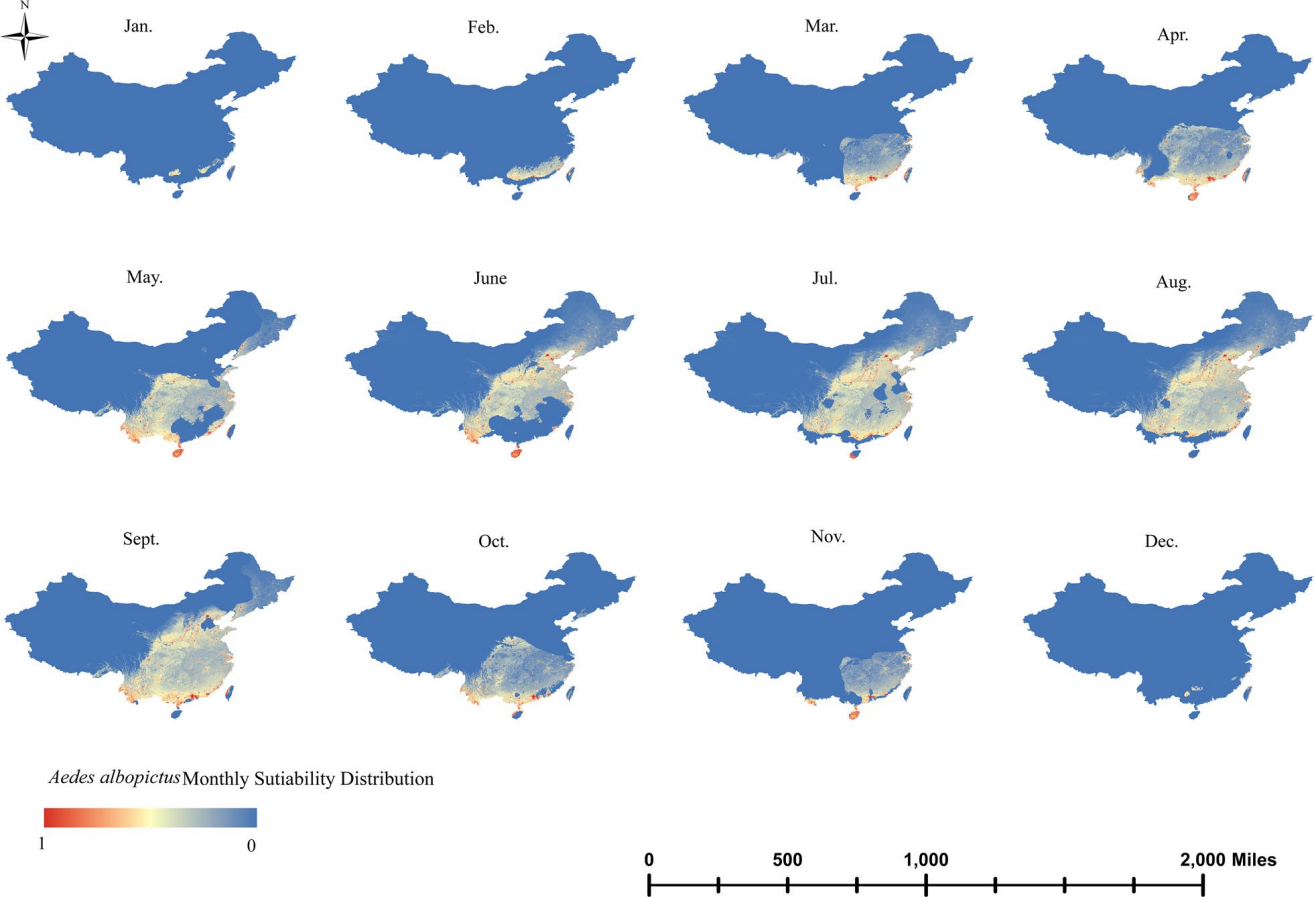
Monthly dynamic distribution range of *Aedes albopictus*

*Aedes albopictus* had a wide distribution in China and persisted over a long time, starting in February and lasting until November. In February and March, *Ae. albopictus* was mainly present in Guangzhou and Fujian, and its distribution gradually expanded to the north as the year progressed. In April, the distribution range of *Ae. albopictus* extended to Yunnan, Hainan, Guangxi, Guangdong, Fujian and parts of Zhejiang. The distribution of *Ae. albopictus* continued to expand and move northwards in May and June, and by July and August, it was present in most areas of southern China. In September, *Ae. albopictus* reached its peak distribution, which—in terms of distribution—was the most suitable month in China; however, Hainan was unsuitable during this time period. The range of *Ae. albopictus* narrowed in October and was mainly concentrated in central and southern China. In November, its distribution range decreased still further, and the range of its presence was approximately the same as in March, but Hainan re-emerged as a suitable distribution area (Fig. 5).

**Fig. 6:**
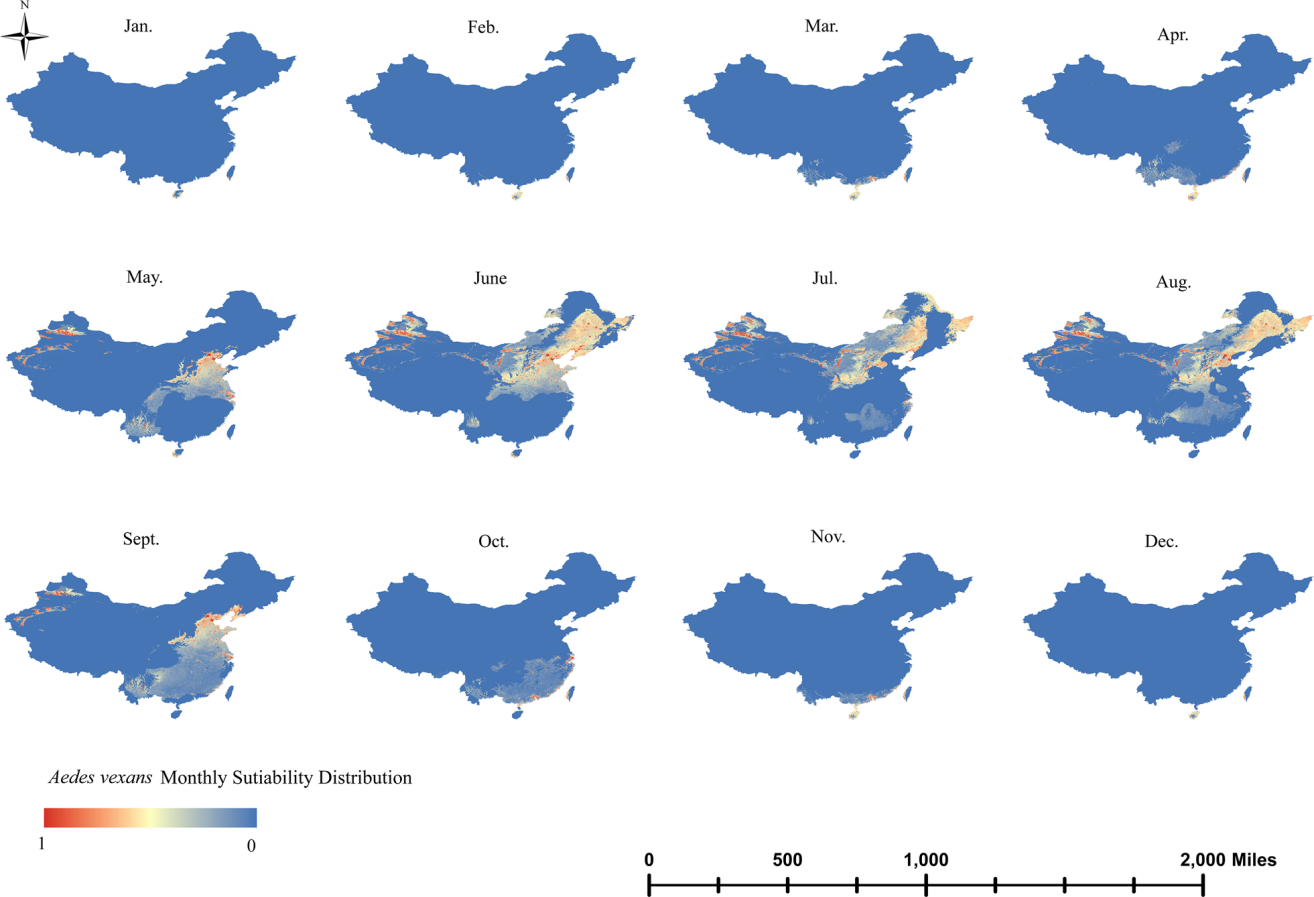
Monthly dynamic distribution range of *Aedes vexans*

*Aedes vexans* was mainly distributed in northern China. In May, *Ae. vexans* appeared in Hebei, the central and western regions of Shandong and parts of Henan and Xinjiang. The range of *Ae. vexans* extended to Jilin, Liaoning and Heilongjiang in June, when its distribution range reached its peak. In July and August, the suitable distribution areas of *Ae. vexans* gradually decreased, and suitable areas were mainly located in the three eastern provinces, Inner Mongolia, Shanxi, Shaanxi, Gansu and Xinjiang. In September, the suitable range for the presence of *Ae. vexans* was further reduced and gradually moved south, mainly distributed in Beijing, Tianjin, Shandong and Jiangsu. *Aedes vexans* was not a dominant mosquito species in the south; thus, its distribution areas in the south were few. Although *Ae. vexans* only occurred in parts of Hainan and Guangdong, it had a persistent presence, and except for July, August and September, this species was well distributed in the other months (Fig. 6).

## Discussion

China has a large territory and encompasses a wide range of climate types. While most areas fall in the temperate zone, some southern regions have a subtropical or tropical climate, northern regions are close to the cold zone, the northwest region has some arid and semi-arid regions and Xinjiang and the Qinghai–Tibet Plateau are characterized by low temperatures throughout the year; this range results in a rich species composition but one that is highly unique in distribution [26, 27]. We collected the distribution records of the three important *Aedes* species in China, each with its own characteristics. *Aedes aegypti*, an invasive mosquito species, is only found in the tropics, but has expanded its range in China since 2000, appearing along the Yunnan border; its presence has led to dengue outbreaks of varying degrees [15]. *Aedes albopictus* has a wide distribution across 25 provinces in China [13] and spans all of the country’s temperature zones [28, 29]. *Aedes vexans* can carry and transmit various mosquito-borne viruses and is a huge potential risk species for the transmission of mosquito-borne viral diseases [30]. Despite its dominance in northern China, studies on its suitability distribution in China are few.

Our study on the importance of variables affecting the distribution of these three mosquito species demonstrated that temperature, precipitation and construction land were the most important factors influencing their distribution, albeit with species-dependent differences. Mosquito density and local climate were significantly correlated [31]. Weather directly regulates the density of adult mosquitoes by temperature and precipitation [12]. Temperature was the absolute factor affecting the distribution of mosquitoes. According to the response curve of temperature change, as the temperature increases, the probability of mosquito distribution also increases, showing a similar linear relationship [32]. The latest research suggests that winter temperature contributes the most to *Ae. albopictus* distribution, followed by summer precipitation [33].

Increased precipitation changes mosquito densities; for example, one study showed that *Ae. albopictus* colonized areas with annual precipitation of > 500 mm when the temperature condition was satisfied [27]. According to the response curves in our study, the probability of distribution of the three *Aedes* species increases with increasing precipitation. High annual temperatures and precipitation favor mosquito activity [34]. Our results show that *Ae. albopictus* were found for the longest time in the hot and rainy southern regions, and were present all year round at times. However, excessive precipitation can also lead to a decrease in the probability of mosquito distribution [35], possibly by affecting the hatching of mosquito eggs and affecting adult mosquitoes [36, 37]. Construction land includes urban, rural and other land developed for human use. According to the response curves, the three *Aedes* species tend to distribute in rural or urban areas where humans gather, with *Ae. aegypti* being more likely to be found in worn-out tires and buckets with stagnant water [38] and in higher abundance in densely populated cities than in other areas [39]. *Aedes albopictus* and *Ae. vexans* were more prevalent in bushes, grasses and livestock sheds near residential areas [18], suggesting that human activity and urbanization are also important factors influencing the spatial distribution of *Aedes*. Interestingly, temperature was not a significant variable affecting the distribution of *Ae. vexans*, possibly due to its distribution characteristics. As the dominant mosquito species in the north of China [40], *Ae. vexans* has a lower temperature limitation and a wider range of suitability for survival; thus, temperature had less influence on *Ae. vexans* than on the other two species.

The optimum temperature and precipitation ranges for the three *Aedes* species were determined based on the equal sensitivity and specificity threshold. Davis et al. [29] defined the optimum temperature ranges for *Ae. aegypti* and *Ae. albopictus* as 17.05 °C to 34.61 °C and 15.84 °C to 31.51 °C, respectively, but these authors also took considered dengue transmission conditions into consideration. Experiments by Brady et al. [41] demonstrated that *Ae. aegypti* were more suitable for survival at about 21 °C, whereas *Ae. albopictus* had a subtle variation in the 20 °C to 30 °C range. Therefore, we adjusted the optimum temperature ranges for both *Aedes* species, whereas the optimum precipitation ranges were determined by an equal sensitivity and specificity threshold. Because of the lack of relevant studies, the optimum temperature and precipitation ranges for *Ae. vexans* were both determined by an equal sensitivity and specificity threshold.

The population dynamics and distribution of *Ae. albopictus* are highly seasonal [42]. Our modeling results indicate that *Ae. albopictus* occurred throughout most of China, including central, southern, and northern China, in the summer (June–September); in only in a few areas of tropical and subtropical China, including Hainan, and a few parts of Guangxi and Guangdong in the winter (December–February); and in southern China in October–November and March–April. This is much the same as the findings of Zheng et al. [26]. *Aedes aegypti* was only found in a few tropical regions of China, with the border areas of Yunnan, the Leizhou Peninsula in Guangdong and Hainan being the main areas of its distribution. The decline in the distribution area of Hainan from July onwards was probably because of a sudden drop in the number of adult mosquitoes due to the effects of excessive precipitation on larval feeding and egg flushing, or to the lack of hosts for feeding on blood [35, 36]. The distribution of *Ae. vexans* was also distinctly seasonal, mainly concentrated from May to September, with the widest distribution range in June. Although the map showed that the southern region of China was also suitable for *Ae. vexans* distribution, no data points were detected there [24].

Our study had three limitations. First, the presence of mosquitoes was mainly based on collected data and was not fully representative of the actual distribution of the whole country; consequently, the range observed may often be smaller than the actual distribution [34]. Second, we did not refine the land cover data and used crude data. Third, the large size of the country and the complex climatic and topographical distribution of China, with large differences in temperature and precipitation from region to region, might have affected model predictions.

Our study enriches previous findings and improves current understanding of *Ae. vexans*; it also provides suitable distribution areas for all three *Aedes* species on a monthly basis. Based on climate and land cover datasets, an ecological niche model was developed to obtain the current optimum distribution areas for each of these three *Aedes* species and to map their monthly dynamic distribution. Our study provides a reference for selecting the best time for mosquito control efforts and mosquito-borne disease prevention and control programs.

## Conclusions

Based on climatic and land cover datasets, maps were created of the current distribution of the three widely distributed mosquito vectors in China—*Ae. aegypti*, *Ae. albopictus* and *Ae. vexans*–and changes in their monthly dynamic distribution range. Based on AUROC values, it is clear that the predictive power and performance of the model were good. *Aedes aegypti* was mainly concentrated in a few tropical regions of China and along the Yunnan border, where it could persist for up to 7 months (April–November). *Aedes albopictus* was widely distributed across most of China, except for the arid and semi-arid regions of northwest China, and had a long duration of distribution, being even present all year round in some areas. *Aedes vexans* was mainly distributed in the temperate regions of northern China, with a shorter distribution period from May to September. As the incidence of mosquito-borne diseases continues to rise, timely detection of key mosquitoes and their eradication in key areas are particularly important.

## Data Availability

The datasets generated during and/or analysed during the current study are available from the corresponding author on reasonable request.
